# A systematic exploration of the micro-blog feature space for teens stress detection

**DOI:** 10.1186/s13755-016-0016-3

**Published:** 2016-04-19

**Authors:** Liang Zhao, Qi Li, Yuanyuan Xue, Jia Jia, Ling Feng

**Affiliations:** Department of Computer Science and Technology, Institute of Data Science, Centre for Computational Mental Healthcare Research, Tsinghua University, 100084 Beijing, China

**Keywords:** Micro-blog, Feature space, Teenager, Stress detection

## Abstract

**Background:**

In the modern stressful society, growing teenagers experience severe stress from different aspects from school to friends, from self-cognition to inter-personal relationship, which negatively influences their smooth and healthy development. Being timely and accurately aware of teenagers psychological stress and providing effective measures to help immature teenagers to cope with stress are highly valuable to both teenagers and human society. Previous work demonstrates the feasibility to sense teenagers’ stress from their tweeting contents and context on the open social media platform—micro-blog. However, a tweet is still too short for teens to express their stressful status in a comprehensive way.

**Methods:**

Considering the topic continuity from the tweeting content to the follow-up comments and responses between the teenager and his/her friends, we combine the content of comments and responses under the tweet to supplement the tweet content. Also, such friends’ caring comments like “what happened?”, “Don’t worry!”, “Cheer up!”, etc. provide hints to teenager’s stressful status. Hence, in this paper, we propose to systematically explore the micro-blog feature space, comprised of four kinds of features [tweeting content features (FW), posting features (FP), interaction features (FI), and comment-response features (FC) between teenagers and friends] for teenager’ stress category and stress level detection. We extract and analyze these feature values and their impacts on teens stress detection.

**Results:**

We evaluate the framework through a real user study of 36 high school students aged 17. Different classifiers are employed to detect potential stress categories and corresponding stress levels. Experimental results show that all the features in the feature space positively affect stress detection, and linguistic negative emotion, proportion of negative sentences, friends’ caring comments and teen’s reply rate play more significant roles than the rest features.

**Conclusions:**

Micro-blog platform provides easy and effective channel to detect teenagers’ psychological stress. Involving comments and responses under the tweet supplement the detection and improves the detection accuracy of 16.8 %.

## Background

The increasingly faster life pace in the competitive society often makes people stressful, especially for teenagers who are not mature enough to deal with psychological pressures properly and effectively. An online survey of 1018 US teens (aged 13–17) made by the American Psychological Association in August, 2013^1^ found that teens were suffering stress in all areas of their lives, from school to friends, work and family, which negatively affects every aspect of their lives, and about 27 % of the teens experienced extreme stress and 55 % experienced moderate stress in the past school year. Adolescence is a critical period for teens’ growth and development. Bearing too much stress without being released timely hurts teenagers physically and mentally, leading to clinical depressions, insomnia, and even suicide. Currently, around 20 % teenagers have psychological illness around the world^2^. According to China’s Center for Disease Control and Prevention^3^, suicide has become the top cause of death among Chinese youth, and excessive stress is considered to be a major factor of suicide. Also in Korea, suicide has become teenagers’ No. 1 killer in the past 2 years^4^.

Hence, it is desirable to be aware of teenagers emotional status, discover their suffered stress, and take effective measures to help teenagers cope with the stress. With the popularity of social networks, many teenagers go to micro-blog for information acquisition, personal interaction, self-expression, and emotion release. Micro-blog has become an open low-cost sensing channel to detect teenager’s emotional status through their tweets.

**Fig. 1:**
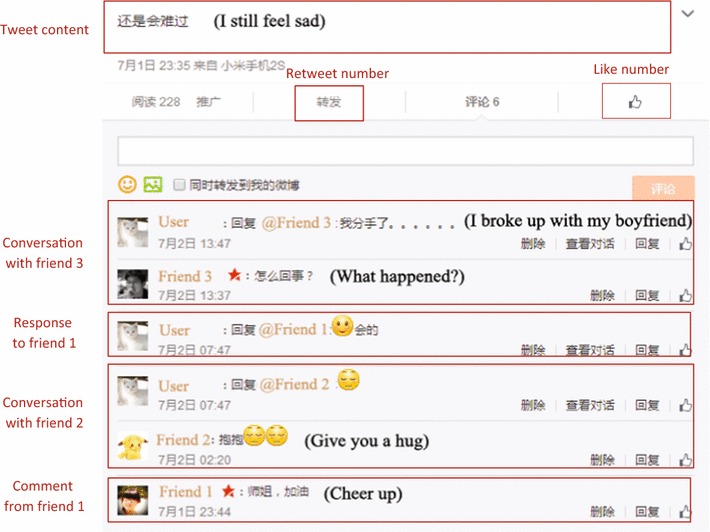
A real tweet example

Recent studies demonstrate the feasibility of tweet-leveled stress and depression detection, since depressed and stressful individuals look micro-blog as a channel for emotional release and interaction [1, 2]. The most closely related work of this paper is [2–7], which designed and implemented a micro-blog platform for sensing and helping ease teens’ stress. A number of tweeting content and tweeting context features were explored for tweet-level teenagers’ stress detection [6]. Detected user’s psychological stress from cross-media micro-blog via a deep convolution network on sequential tweeting time series in a certain time period. Considering a tweet is limited to 140 characters, which may not be long enough for teens to express their stress categories and stress levels, [7] incorporated social interactions between teens and their friends under each tweet in stress detection. Due to the topic continuity from the tweeting content to the follow-up comments and responses between the teen author and his/her friends, [7] combined the contents of comments and responses under the tweet to supplement the tweeting content. Take a real tweet for example. From the tweeting content “I still feel sad.” in Fig. 1, it is hard to recognize the stress category. However, from the comments and responses under the tweet, sentence “I broke up with my boyfriend.” reveals this teen has an affection stress.

In this study, we extend the previous work on stress detection with a systematic examination of the micro-blog feature space for teens stress detection. A 4-dimensional feature space, comprised of four kinds of features [tweeting content features (FW), posting features (FP), interaction features (FI), and comment-response features (FC) between teenagers and friends], is presented for stress category and stress level detection. Their impact on detection performance is investigated based on entropy and conditional entropy. Our performance study shows that all the features in the feature space positively affect stress detection. Among the features, linguistic negative emotion, proportion of negative sentences, friends’ caring comments and teen’s reply rate play more significant roles than the rest features.

The remainder of the paper is organized as follows. We discuss some closely related work in “Related work” section, and present our method for constructing the micro-blog feature space for teens stress detection in “Methods” section. We report our user study in “Experiments” section. Finally we conclude the paper in “Discussion” section.

## Related work

### Emotion analysis on social networks

Computer-aided sentiment analysis and applications on social networks have drawn much attention in recent years [8–14]. Most of the work intended to classify tweets emotion into positive, neutral, or negative; or happy, angry, fear, sad, surprised, or disgusted [15]. Leveraging content features such as text-based linguistic attributes and visual factors such as emoticons and images, [16] developed a system called Moodlens to do sentiment analysis for Chinese Weibo tweets. It divided human’s sentiment states into four types (i.e., angry, disgusting, joyful, and sad), and then applied a fast Naive Bayes classifier to tweet-level sentiment analysis [17]. Built a four-leveled emotion hierarchy from specific emotions (e.g., sad, surprised, etc.) to general ones (e.g., neutral or emotional) and applied such hierarchical emotions to sentiment analysis upon Chinese Weibo tweets. Emoticons and Part-Of-Speech (POS) features were also extracted to assist emotion classification [18]. Extracted text feature vectors with domain sentiment dictionaries and classified the positive or negative emotion of a tweet using the classic SVM method. Given a query term, [19] analyzed its positive, neutral, or negative emotion tendency through the emotions of all its related tweets and adopted unigram, bigram, and POS as features for emotion classification [20]. Extracted sentiment words, internet slangs, and emoticons in tweets, and used LibSVM to identify positive or negative emotions expressed in topics, not the tweets. Forwarding, commentaries, and sharing under the tweet are involved to further optimize the result [21]. Analyzed emoticons as well as lexical features (e.g., segmented Chinese words, Chinese characters, and higher order n-grams) and classified the emotions of tweets into the classical six categories (i.e., happy, angry, fear, sad, surprise, disgusted) [22]. Proposed a novel method for text-based emotion classification through the extracted emotion cause events from micro-blog posts. It designed a rule-based sub-system to detect and extract events from the original posts based on the common social network characteristics and carefully-generalized linguistic patterns [23]. Utilized 50 twitter tags and 15 smileys as sentiment labels to automatically identify diverse sentiment types of short texts [24]. Calculated the emotional intensity with the three levels of words, sentences, and documents on micro-blog based on the HowNet knowledge base. With a set of typed dependency polarity pattern rules and phrase-level analysis, [25] presented a polarity prediction model to predict the sentiment polarities of sentences [26]. Exploited the Karhunen–Loeve Transform (KLT) and average distances of positive and negative texts to detect sentiment similarity, and further analyzed similar Chinese micro-blog accounts [27]. Modeled the emotion classification using SVM from writer/reader perspectives upon the Plurk social network. Chinese character bigrams are leveraged as text features. In [27], social relations between users (i.e., how often the two users interact, how often a user posts messages or makes replies), individual user behavior (i.e., subjective positive or negative tendency of the user), as well as the relevance degree between the posts and attached replies were exploited as non-linguistic features [28]. Conducted sentiment analysis of Chinese Sina Weibo based on semantic sentiment space model. From emoticon sentences on Yahoo! blogs, user’s emotions were detected [29]. Four approaches, including topical approach, emotional approach, retrieval approach, and lexicon approach, were designed to calculate the emotional score of each word. The emotion class of the log was then determined by accumulating the emotional score of words.

### Stress-related analysis on social networks

Recent studies demonstrate the feasibility of tweet-leveled stress and depression detection, since depressed and stressful individuals take micro-blog as a channel for emotional release and interaction [1, 2, 4]. For example, [30, 31] analyzed users’ twitting behaviors to measure their depression risks, and found out that social media contains useful cues in predicting one’s depression tendency [32]. Built a depression detection model based on a sentiment analysis method on micro-blog. Looking into the contents and temporal features of users’ BBS posts, [33] tried to detect depressed users through a supervised learning approach.

Focusing on the four main kinds of stress (academic, affection, interpersonal and self-cognition) that teenagers usually encounter, [2] extracted different features from teenagers’ tweets, including negative emotion words, negative emoticons, unusual post time and post frequency, etc., and tried five classifiers (Naive bayes, support vector machines, artificial neural network, random forest, and gaussian process classifier) to learn the potential stress category and corresponding stress level (none, very weak, weak, moderate, strong, and very strong) revealed from each tweet according to the stress detection result, [3] designed and implemented a micro-blog based system, aiming to provide stressful teens timely intervention support like recommending jokes, funny pictures, and encouraging stories, suggesting some simple breathing and muscle relaxation exercises, guiding the teenagers to write down something for self-expression, or notifying teens’ guardians when the stress is serious in the worst case.

Combining the content features and multimedia information such as color theme and brightness of the images posted in tweets, [5] presented a deep sparse neural network to detect tweet-level stress for arbitrary micro-blog users (not teenagers specifically). However, [5] only detected whether the user suffers from stress, but did not quantitatively measure the stress level. Furthermore, [6] detected user-level stress from cross-media micro-blog via a deep convolution network on sequential tweeting time series in a certain time period. From each single tweet, [2, 5] extracted a category-independent feature vector. They detected different stress categories in the tweet by analyzing categorical words, and all the detected stress categories share the same stress level in a single tweet. To overcome the limitation of short text in a tweet, [7] further improved the above tweet-based adolescent stress detection work [2, 5, 6] by augmenting each tweeting content with friends’ comments and user replies. Other derived information like user’s reply rate, caring comment rate, and average interaction depth were also exploited in the examination of user’s psychological status.

## Methods

We consider four typical stress categories (i.e., academic, affection, interpersonal, and self-cognition) that teenagers frequently experience, as well as three stress levels (i.e., light, moderate, strong) in the study. Let category = {“academic”, “affection”, “interpersonal”, “self-cognition”, “unknown”}, and level = {0, 1, 2, 3}, corresponding to none, light, moderate, strong stress level.

From a teen’s tweet, we aim to sense teen’s possible stress categories and corresponding stress levels, that is, stress (tweet) = < L (academic), L (affection), L (interpersonal), L (self-cognition), L (unknown) >, denoting the sensed stress level in the stress category academic, affection, interpersonal, and self-cognition, respectively, where L (unknown) denotes the level of the stress whose category is unknown from the tweet. A tweet is called *a stressful tweet*, if there exists a none-zero stress level in {L (academic), L (affection), L (interpersonal), L (self-cognition), L (unknown)}, and otherwise, it is called *a non*-*stressful tweet*.

**Fig. 2:**
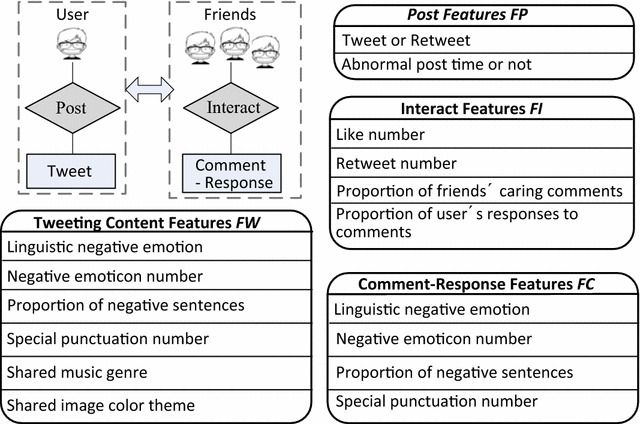
Micro-blog feature space for sensing teens stress

A four-dimensional micro-blog feature space F = (FP, FW, FI, FC) (Fig. 2) is explored for stress detection, where

1. FP depicts user’s posting behavior;
2. FW depicts user’s posting content;
3. FI depicts user and its friends’ social interaction;
4. FC depicts friends’ comment and user’s response contents under the tweet.

### User’s posting features FP

User’s posting features FP contain two elements.

*FP1(.): Tweeting or retweeting TweetOrRetweet (tweet).* This feature distinguishes whether the tweet is originally authored by the user or is a retweeted one made by someone else. Investigating the tweet’s originality lies in the observation that in most cases, users express themselves and record their daily life by originally composed tweets, while the retweeted posts are usually news, jokes, bookmarks, advertisements, or contents holding the same opinion. Thus, compared with a retweeted post, an original tweet can better reveal user’s stressful emotion. TweetOrRetweet (tweet) = 1 if the post is an original tweet, and 0 otherwise.

Notation (.) indicates that this feature element applies to all the category C∈category

*FP2(.): Abnormal post time or not AbnormalPostTime (tweet).* Teenagers usually follow a rather stable schedule with regular semesters and holidays. Their activities on micro-blog are thus rather stable. Abnormal post time may possibly imply their abnormal status. Considering different daily routines of teenagers during holidays and non-holidays, we distinguish three schedules, i.e., holidays (winter holiday in January and February, and summer holiday in July and August), non-holiday weekends, and non-holiday-weekdays, respectively. Each day is equally divided into 24 time intervals, representing 24 h.

To measure the deviation of a teen’s post time from the normal one, we examine teen’s historic post time series, and calculate the possibility that a tweet posted within the same time period historically is a stressful tweet. Assume a user posts a tweet within the k-th time interval of a day following schedule s, the abnormal possibility of this tweeting time period is: AbnormalPostTime (tweet) = SNum (k, s)/Num (k, s),where SNum (k, s) denotes the number of user’s stressful posts at the k-th interval of a day in schedule s, and Num (k, s) denotes the total number of tweets posted at the k-th interval of a day in schedule s.

### User’s tweeting content features FW

Compared to other sensory means, one advantage of using micro-blog to sense human users stress lies in its stress category detection based on linguistic contents.

#### Identification of stress categories from each tweet

Due to the limited textual content in each tweet, we detect the stress category and corresponding stress level in the category at the finest granularity-clause. From each clause, we extract words using the open source Chinese word analyzer tool Ansj (http://www.ansj.org/), and then identify categorical words according to the teenagers stress-related lexicons TSL [2] and the widely used psychological dictionary LIWC [34]. For example, school and homework are both categorical words in the academic stress category. It is possible that multiple categorical words may be discovered from the clause, and label the clause categorically.

**Fig. 3:**
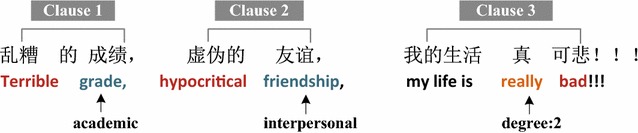
An example tweet where the *red words* are negative emotion words, the *green* ones are categorical words and the *orange* ones are degree adverbs

If no categorical words appear in the clause, we firstly check other clauses in the same sentence, and take the sentence’s categorical words as this clause’s. If the whole sentence does not contain any categorical word, we then check next sentence, and take the next sentence’s categorical words as the clauses. If no categorical words are found throughout the whole tweet, then the stress category will be labeled as “unknown”. Take the example tweet in Fig. 3 as an example, there is no categorical words detected in clause 3 and we estimate the categories by that of clause 1 and 2. Obviously, the categories of clause 3 are academic and interpersonal.

#### Instantiation of tweeting content features FW for category-aware stress level detection

For each stress category C∈category, we instantiate the following six tweeting content features by examining all the clauses associated with category C.

*FW1(C): linguistic negative emotion NegEmotion (tweet, C).* It is the amount of linguistic negative emotion expressed in the tweet in category C, measured by both the number of negative emotion words and the emotional degree reflected by the degree adverbs which modify the negative emotion words. We check the pair of degree adverb and the modified negative emotion words in the clauses in category C according to TSL and LIWC. Three degrees (light, moderate, strong, valued one, two, three, respectively) of degree adverbs are considered according to the TSL lexicon. For a negative emotion words with no degree adverb modified, its emotional degree is considered as light (valued one). Particularly, when computing the linguistic negative emotion, the following three semantic rules are applied.

- Negative emotion words bounded with single negator express positive meaning and are ignored, while positive emotion words bounded with single negator is considered as negative ones.
- Double negators represent positive meaning.
- When a degree adverb is bounded with a negator, its degree will be converted. For example, a tweet says “I’m not so happy”, in which the adverb degree of “so” is three (strong), but with negator “not” the actual degree converts into one (light), equally with “I’m a little unhappy”.

We take the sum emotion amount of all the degree adverb and the modified negative emotion words pairs in category C as NegEmotion (tweet, C). For example in Fig. 3, the tweet says “Terrible grade, hypocritical friendship, my world is really bad!!!”, then we calculate NegEmotion (tweet, academic) = 1 + 2 = 3, and NegEmotion (tweet, interpersonal) = 3.

*FW2(C): negative emoticon number NegEmoticonNum (tweet, C).* Emoticons are usually used in tweets to supplement natural language expressions. Negative emoticons tend to express users bad emotion. NegEmoticonNum (tweet, C) is the sum of negative emoticon numbers in the involved clauses associated with C.

*FW3(C): proportion of negative sentences NegSentenceRatio (tweet, C).* It is the ratio of sentences containing negative emotion word(s) or negative emoticons in category cover the total number of sentences in tweet. A bigger proportion of negative sentences indicates a stronger stress in category C.

*FW4(C): Special punctuation number PunctuationNum (tweet, C).* Duplicate use of some special punctuation marks like exclamation mark “!”, question mark?, ellipsis mark…, and Chinese full stop marks ◦◦◦ expresses users some emotion. PunctuationNum (tweet, C) is the total number of these special punctuation marks that are used in the clauses associated with C. In Fig. 3, PunctuationNum (tweet, academic) = 3.

*FW5(.): shared music genre NegMusic (tweet).* Besides the linguistic text, users may post other media information like music and images within a tweet. The genre of the shared music reflects one’s emotion more or less. A stressful user with a low mood tends to share sad music in his/her tweet. We collect and continuously maintain a title pool of sad music, and examine whether a shared music in a tweet is sad or not based on the pool. So far, the pool contains 315 popular pure and song music. A further personal music pool is to be developed in the near future. NegMusic (tweet) = 1 if the music is sad, and otherwise NegMusic (tweet) = 0.

*FW6(.): shared image color theme NegImage (tweet).* Similar to sad music genre, the color of the posted image carries certain emotions According to psychology and art theories [35]. We measure the negative emotion of an image through a 2-dimensional color theme (i.e., warm or cool and hard or soft) using the technique [36]. For a tweet containing multiple images, we take the mean color theme of these images.

### User-friends interaction features FI

Apart from extracting tweeting content features from a tweet, we also examine the social interaction incurred between the user and friends for information supplement.

*FI1(.): Like number LikeNum (tweet).* People usually like to put a like seal on a tweet to show their positive emotion or attitude to the tweet. Compared with those positive tweets, stressful tweets (negative tweets) obtain much less likes, even no likes at all. LikeNum (tweet) is the total number of likes that tweet receives from the teen’s friends.

*FI2(.): retweet number RetweetNum(tweet).* Also, people seldom retweet (forward) quite personal tweets of other users, especially for stressful tweets. RetweetNum (tweet) is the total number of retweets that tweet receives from the teen’s friends.

*FI3(.): proportion of caring comments CareCommentRatio (tweet).* Among all the comments from friends, we check how many of them belong to caring, comforting, or encouraging comments like “What’s up?”, “Don’t worry.”, “Everything will be OK.”, or hug emoticon. We construct a lexicon containing over 200 caring phrases and emoticons for caring comments identification. CareCommentRatio (tweet) is the ratio of caring comments versus the total number of comments underneath tweet. A higher proportion of caring comments denotes a bigger probability that the tweet is stressful.

*FI4(.): proportion of user’s responses to comments ResponseRatio (tweet).* ResponseRatio (tweet) is the ratio of user’s response number versus the total number of comments regarding tweet. Psychological study^5^ shows that people with a negative emotion tend to behave abnormally, being less active on social networks.

#### User-friends comment-response features FC

Considering a tweet is limited to 140 characters, it may be too short to provide sufficient information to figure out one’s stress. Also a teen may not always speak out his/her stress in a brief tweet. However, underneath the tweet, friends’ comments and teen’s responses to these comments may supply some hints on teen’s stress categories and stress levels.

Let friends be a set of friends that comment tweet, and resp (tweet, f) (where f ∈ Friends) be a set of linguistic teen’s responses to the same friend f’s comments. We view resp (tweet, f) as a kind of new tweet, and each response as a sentence. When there are multiple consecutive responses to friend f, we combine and collapse them into one response. In a similar way like tweet, for each stress category C∈Category, we identify categorical words from resp (tweet, f) and compute the following four features.

*FC1 (f, C): Linguistic negative emotion NegEmotion (resp (tweet, f), C).*

*FC2 (f, C): Negative emoticon number NegEmoticonNum (resp (tweet, f), C).*

*FC3 (f, C): Proportion of negative sentences NegSentenceRatio (resp (tweet, f), C).*

*FC4 (f, C): Special punctuation number P unctuationNum (resp (tweet, f), C).*

If no categorical words are discovered throughout resp (tweet, f), we examine f’s comments, and take their categorical words as the responses’ ones.

In this way, from each resp (tweet, f), we obtain the above four features. Since the comments and responses under the tweet may not definitely talk about the same thing with the tweet itself, to avoid extra noise, we combine them and enhance the very original tweet’s content features FW1, FW2, FW3, and FW4 in the following two cases.

Assume CS is the set of categories detected in tweet, and for each C∈CS, [Case 1 (C ≠ unknown)] combine the features in the same category detected in resp (tweet, f) to enhance the stressful expression by

FWC1 (C) = FW1 (C) + ∑_f∈Friends_ FC1 (f, C);

FWC2 (C) = FW2 (C) + ∑_f∈Friends_ FC2 (f, C);

FWC3 (C) = (NumNegSent (tweet) + ∑_f∈Friends_ NumNegSent (resp (tweet, f)))/.

(NumSent (tweet) + ∑_f∈Friends_ NumSent (resp (tweet, f))), where NumNegSent (.), NumSent (.) are the number of negative sentences and the total number of sentences of content “.”, respectively;

FWC4 (C) = FW4 (C) + ∑_f∈Friends_ FC4 (f, C);

[Case 2 (C = unknown)] (that is, no explicit stress category is detected in tweet),

for each category (C’ ≠ unknown)∧(C’∈CSR), where CSR is the category set detected in resp(tweet, f), we estimate the potential stress categories expressed in the tweet by the categories detected in resp(tweet, f), and keep the original feature values under C, instead of combining the feature values in resp(tweet, f), since we cannot tell whether tweet of an unknown category is surely related to resp(tweet, f) in content.

FWCi (C’) = FWi (unknown) (i = 1, 2, 3, 4).

For the example in Fig. 1, there is a negative emotion words sad in the tweet content (i.e., FW1 (tweet, unknown) = 1). From resp (tweet, f3) we detect the category affection, then we get the feature FWC1 (affection) = 1.

For each stress category C∈Category, we formulate a category-aware feature vector as F(C) = (FP (.), FI (.), FWC (C)). For a single tweet, all the stress categories share the same feature values of FP and FI.

## Experiments

We invite 36 high school students (15 males and 21 females, aged between 15 and 17) in Shanxi Province, China to participate in our user study. We crawl their 21,648 tweets from Tencent Weibo platform (one of the biggest Chinese micro-blog platform, http://www.t.qq.com/) in the period of 2013/1/1 to 2015/5/1. Each participant posted around 601 tweets on average. We then invite the participants to examine their own tweets one by one, and label their stress categories and corresponding stress levels by recalling their previous experiences. These annotations are taken as the ground truth.

Table 1 illustrates the proportions of various stressful tweets in our experimental data set. The sum proportion of stressful tweets in the four deterministic stress categories is 6.33 %, bigger than the proportion of stressful tweets (5.26 %). This verifies our assumption that a tweet may exhibit teen’s multiple stress categories.

**Table 1:** Proportions of stressful tweets in the user study

	Number of tweets	Proportion of tweets (%)
Total number of tweets	21,648	100
Number of stressful tweets	1139	5.26
With academic stress	439	2.03
With affection stress	385	1.78
With interpersonal stress	342	1.58
With self-cognition stress	203	0.94

From each of the total 21,648 tweets, we extract and initiate a feature vector corresponding to each stress category C∈Category. In total, we get 108,240 (= 21,648*5) feature vectors. 10-fold cross validation upon all the feature vectors is leveraged. We then apply four different classifiers, i.e., Naive Bayes, Logistic Regression, Random Forest, and SVM, to perform the single-tweet based stress detection over the feature space in each stress category. Precision (measuring how many detected stress levels exactly match the participants’ annotations) and recall (measuring how many stress levels are not detected while the participants annotate the tweets as stressful) are used to evaluate the performance. Since the participants did not mark an unknown stress category to a tweet, if we detect a stress level in an unknown stress category, we attribute it to the stress categories that the participant marks and seek the maximal matching degree.

### General performance of stress detection in the four stress categories

As presented in Table 2 shows the stress detection performance of the four classifiers. All the classifiers perform poorly in the self-cognition stress category due to data sparsity. This may be because the tweets in the self-cognition category are the minimum and occupy only 0.94 % (Table 1). In all the stress categories, SVM performs the best among the five classifiers, with the average F-measure over 70 %, which is 16.7 % better than NB, and 2.9 % better than random forest and logistic regression. Thus, in the following experiments, we choose SVM as the classifier for stress detection. Compared with the other stress categories, affection and self-cognition stress detection perform a little poorer. This may due to a wider expressiveness in language of these two kinds of stress, while academic stress (mainly including study and school) and interpersonal stress (mainly including conflicts, quarrels with others for teenagers) are possibly a smaller domain with less language ambiguity.

**Table 2:** Performance of category-dependent stress detection

Stress category	Naive bayes			Logistic regression			Random forest			SVM		
	Prec.	Rec.	F-ms.	Prec.	Rec.	F-ms.	Prec.	Rec.	F-ms.	Prec.	Rec.	F-ms.
Academic	0.56	0.69	0.62	0.72	0.71	0.72	0.63	0.66	0.65	0.71	0.71	0.71
Affection	0.50	0.69	0.58	0.69	0.63	0.66	0.72	0.69	0.71	0.70	0.65	0.67
Inter-personal	0.56	0.70	0.63	0.72	0.71	0.72	0.74	0.73	0.73	0.78	0.75	0.76
Self-cognition	0.52	0.64	0.58	0.62	0.62	0.62	0.64	0.63	0.64	0.68	0.67	0.67
Avg.	0.54	0.68	0.60	0.69	0.67	0.68	0.68	0.68	0.68	0.72	0.69	0.70

### Performance of stress level detection in the four stress categories

**Fig. 4:**
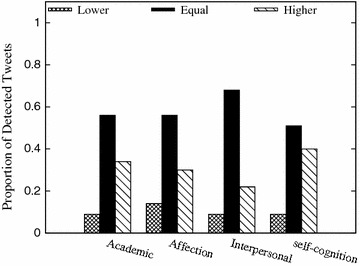
Performance of stress level detection

For the tweets which are annotated as stressful by the users and also have nonzero detected stress levels, we compare their stress level differences using detected level lower than, equal with, and higher than what the user annotated. Figure 4 shows that for the four stress categories, tweets with accurately detected stress levels occupy more than 50 % of the whole stressful tweets, and around 10 % tweets are detected with unexpected lower stress levels. It illustrates that our detection method based on the comprehensive feature space will not miss most of the heavily stressful tweets.

### Performance of stressful-or-not detection in the four stress categories

**Fig. 5:**
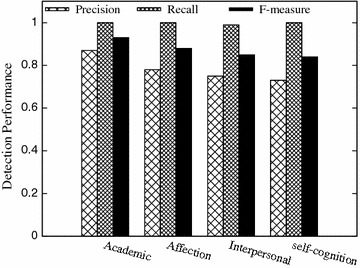
Performance of stressful-or-not detection

Ignoring fine-grained stress levels (none, light, moderate, and strong), we consider stressful-or-not detection. Tweets with non-zero stress level of each category is regarded as stressful in the category, otherwise non-stressful. Figure 5 illustrates the precision, recall, and f-measure of our detection result, where the average F-measure is over 80 %. Detection of academic stress has the best performance, and detection of self-cognition stress has the worst performance. This is obvious as the data set is unbalance with most stressful tweets talking about study and school and the least addressing self-cognition related issues. This is coincident with the teenagers’ daily activities.

### Feature impact

We leverage information gain to investigate the impact of different micro-blog features on stress detection based on entropy and conditional entropy. Information gain represents the change of information amount involving feature F. A bigger information gain suggests a bigger significance of the feature. Assume L∈Level, C∈Category, feature F has m different values, and Fj denotes the j-th value of F. Prob (.) represents probability and H (.) is the entropy. InfoGain (L, C|F) = H (L, C) − H(L, C|F), where

$$H\left( {L,C} \right) = - \mathop \sum \limits_{k = 1}^{|Level|} \left( {Prob\left( {L = L_{k} ,C} \right) log Prob(L = L_{k} ,C)} \right)$$

$$H\left( {L,C|F} \right) = \mathop \sum \limits_{J = 1}^{m} \left( {Prob\left( {F = F_{J} } \right)H(L,C|F = F_{J} )} \right)$$

$$H\left( {L,C|F_{J} } \right) = \mathop \sum \limits_{k = 1}^{|Level|} \left( {Prob\left( {L = L_{k} ,C|F = F_{J} } \right) log Prob(L = L_{k} ,C|F = F_{J} )} \right)$$

**Fig. 6:**
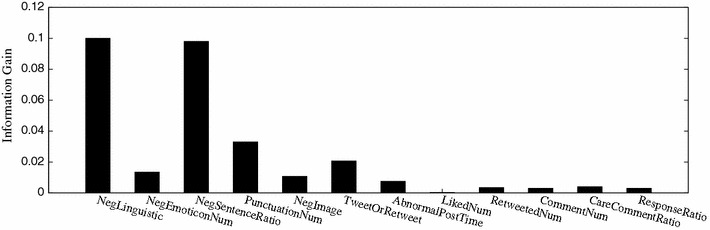
Information gains of different features on the data set

**Fig. 7:**
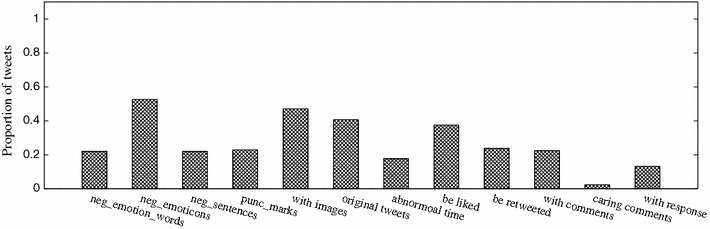
Proportion of tweets containing different features on the data set

Figure 6 shows the average information gain of each micro-blog feature in the detection of stress categories and stress levels from the data set. Feature image color theme contains two dimensional values (warm or cool and hard or soft). We take their average information gain to measure the negative image emotion degree. From the result presented, we can see that all the listed features bring positive impact on stress detection, and the top-3 features are linguistic negative emotion FC1 (f, C), proportion of negative sentences FC3 (f, C), and special punctuation number FC4 (f, C), extracted and computed based on the tweeting and comment-response contents. This coincides with the fact that explicit statement is the most direct indicator of stress expression. Comparatively, the information gain of feature proportion of caring comments FI3 (.) is only 0.002. The reason for its low information gain is due to the sparse and unbalance of the data set, where only 2.4 % tweets in total contain caring comments. Figure 7 gives the distribution of different features on the total data set.

**Fig. 8:**
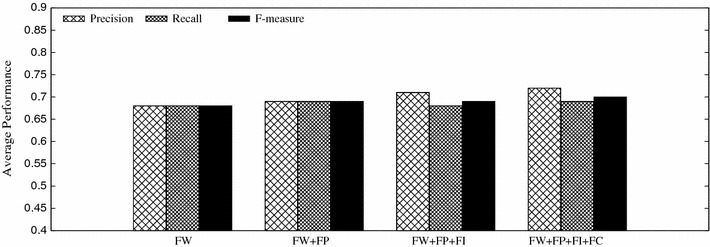
Detection performance of different feature combinations on the data set

We further compare the detection performance under different combinations of tweeting content features (FW), posting features (FP), interacting features (FI), and comment-response features (FC). Figure 8 shows that different feature combinations make little difference upon the total data set. This is attributed to data imbalance that only a small part of tweets are attached with social interactions (e.g., comments and responses) and tweeting content features dominate the detection result.

**Fig. 9:**
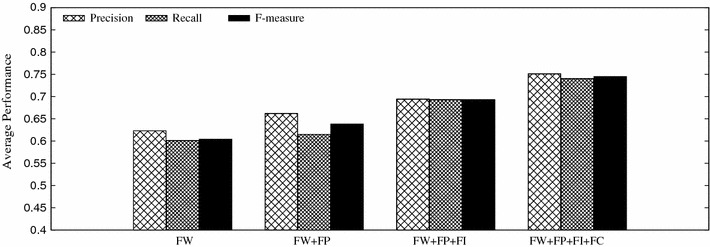
Detection performance of different feature combinations on the data subset containing tweets with comments

To eliminate data imbalance, we also examine the performance of different feature combinations on the data subset, containing tweets with comments. It is interesting to see that this time, features of social interaction and comment-response play positive roles in stress detection, as illustrated in Fig. 9. Relying only on tweeting contents FW cannot provide enough information. Together with the posting behavior features (FW + FP), the performance improves around 5.63 % than merely FW. After adding social interaction and comment-response features (FW + FP + FI + FC), the average F-measure reaches 75 %, outperforming FW by 23.3 %, and (FW + FP) by 16.8 %. This verifies the significance of social interactions in the micro-blog feature space construction.

## Discussion

We consulted a psychological doctor in the Affiliated Yuquan Hospital of Tsinghua University, who evaluated the importance of this research highly. According to him, mental disorder is always accompanied with some abnormal daily behaviors. Thus, treatment and prevention of mental diseases could be carried on in different levels. Take school students for example. The first level is to check some academic performance like examination pass-or-not. The second level is to examine some physical and social activities like tweeting on social networks. When some abnormal signals are revealed, the last level is to go to a doctor.

While the experimental result is promising, a number of issues remain to be addressed in the near future. In our approach, we calculate the feature value of AbnormalPostTime (tweet) based on teens historical posting behavior. This brings a cold start problem that if a teen just starts posting tweets, we cannot judge whether a posting time is abnormal or not. Considering teenagers usually follow regular schedules (e.g., 8:00 a.m.–6:00 p.m. for school, homework every night, relaxed weekends, etc.), one possible solution is derive the feature value based on teen’s friends or other teens with a similar background.

## Conclusions

In this paper, we analyze and extract four kinds of micro-blog features, including teen’s posting behavior features, tweeting content features, social interaction behavior features, and comment-response contents under the tweet between the teen and his/her friends, for teenagers’ stress detection. We then apply classic classifiers to detect teen’s stress levels in possibly multiple stress categories from a tweet. Our experimental results demonstrate all the features extracted positively impact the stress detection performance. Among the features, linguistic negative emotion, proportion of negative sentences, special punctuation number, proportion of caring comments, and proportion of user’s responses to comments, extracted and computed based on the tweeting and comment-response contents play significant roles. We are currently integrating multiple sources to enhance stress detection.

## Footnotes

1 http://www.usatoday.com/story/news/nation/2014/02/11/stress-teens-psychological/5266739.

2 http://www.learning.sohu.com/s2012/shoot/.

3 http://www.theweek.com/article/index/252199/the-rise-of-youth-suicide-in-china.

4 http://www.learning/sohu.com/20130402/n371458123.shtml.

5 http://www.pewinternet.org/files/2015/01/PI_Social-media-and-stress_0115151.pdf.
